# Oral Probiotics Alleviate Intestinal Dysbacteriosis for People Receiving Bowel Preparation

**DOI:** 10.3389/fmed.2020.00073

**Published:** 2020-02-28

**Authors:** Xiaorong Deng, Huakai Tian, Rong Yang, Yiwen Han, Kehong Wei, Cihua Zheng, Zhaoxia Liu, Tingtao Chen

**Affiliations:** ^1^Department of Gastrointestinal Surgery, The Second Affiliated Hospital of Nanchang University, Nanchang, China; ^2^National Engineering Research Center for Bioengineering Drugs and the Technologies, Institute of Translational Medicine, Nanchang University, Nanchang, China; ^3^Department of Obstetrics and Gynecology, The Second Affiliated Hospital of Nanchang University, Nanchang, China

**Keywords:** bowel preparation, probiotics, high-throughput sequencing, *Proteobacteria*, *Firmicutes*/*Bacteroidetes*

## Abstract

**Background:** Bowel preparation is necessary for successful colonoscopy, while it can seriously affect intestinal microbial composition and damage the intestinal mucosal barriers in humans.

**Methods:** To figure out whether probiotics can sustain intestinal homeostasis and guard people's health, the probiotic drug of *Bifidobacterium* Tetragenous viable Bacteria Tablets (P group, *n* = 16) or placebo (C group, *n* = 16) was used for volunteers receiving bowel preparation, and high-throughput sequencing method was applied to monitor their intestinal microbial changes.

**Results:** The present results suggested that bowel preparation obviously reduced the intestinal microbial diversity, while taking probiotics significantly restored it to normal level. In addition, probiotics sharply reduced the abundance of pathogenic *Proteobacteria*, and obviously lowered the ratio of *Firmicutes/Bacteroidetes* compared with control group at phylum level (*P* < 0.05). And probiotics markedly decreased the abundance of pathogenic *Acinetobacter* and *Streptococcus*, while greatly enriched the relative abundance of beneficial bacteria *Bacteroides, Roseburia, Faecalibacterium*, and *Parabacteroides* at genus level (*P* < 0.05).

**Conclusion:** Probiotic drugs, e.g., *Bifidobacterium* Tetragenous viable Bacteria Tablets, can be used to restore intestinal dysbacteriosis caused by bowel preparation, and reduce side effects during colonoscopy.

## Introduction

Colonoscopy is the preferred method to evaluate the intestinal health of most patients and it is the gold standard for colorectal cancer diagnosis, which can clearly discover intestinal lesions (1). Before colonoscopy, bowel preparation is commonly used to ensure that no residue remains in the intestinal wall to affect the examination process and results via utilizing adjusted diet and related drugs, and the adequacy of bowel preparation can directly affect the final effect of colonoscopy (2). At present, polyethylene glycol (PEG) is widely used for intestinal cleaning before colonoscopy due to its effectiveness and extensive acceptability (3).

As we know, the intestinal microbiota is important to sustain human health. Under physiological conditions, symbiotic physiological anaerobic bacteria, symbiotic conditional pathogenic bacteria and other harmful bacteria co-exist in the intestinal tract in a stable proportion. However, when intestinal microbiota alters or the proportion of intestinal microbiota is out of balance, corresponding pathophysiological changes will occur (4). During the bowel preparation procedure, large quantity of liquid enters the intestinal tract and considerably disrupts the environment of the normal intestinal cavity, and taking laxatives can enhance intestinal dyskinesia and intestinal peristalsis, which makes bacteria cannot adhere to intestinal mucosa (5). Moreover, the large amount of oxygen brought by bowel preparation in intestinal environment heavily reduces the number of anaerobic bacteria, and promotes the growth of aerobic bacteria, resulting in intestinal microbial disorder (6).

Probiotics are “living microorganisms that can have beneficial effects on the host when ingesting sufficient doses” (7), previous studies indicate that probiotics play an active role in a variety of human diseases, including irritable bowel syndrome, inflammatory bowel disease, and colon cancer (8, 9). Our previous studies indicated that probiotic preparations have important effects in reducing inflammatory response after gastrostomy and improving gastrointestinal symptoms in post-operative patients (10), and probiotic preparations also significantly alleviated oral mucosa inflammation caused by radiotherapy in patients with nasopharyngeal carcinoma (11). Although attention has been paid on the side effects of bowel preparation, e.g., imbalance of intestinal microbiota and damages to intestinal mucosa in academic circles, little work is done to reduce side effects of bowel preparation using probiotic supplement.

In the present study, the clinical probiotic drug of *Bifidobacterium* Tetragenous viable Bacteria Tablets was used to evaluate its effect on volunteers receiving bowel preparation, and high-throughput sequencing was applied to assess whether probiotics had positive effects on intestinal microbiota disorder caused by bowel preparation.

## Materials and Methods

### Ethics Statement

The present study was approved by the Institutional Review Boards of the Second Affiliated Hospital of Nanchang University (Nanchang, China). Patients provided written informed consent for sample collection. The project has also been registered and approved by the China Clinical Trial Registration Centre (ChiCTR1900022539).

### Study Design and Patient Enrolment

The trial was conducted at the Second Affiliated Hospital of Nanchang University in China between December 2018 and November 2019. Thirty-two subjects (29 males, 3 females), with an average age of 51 y (range 30–70 y), height of 1.66 m, weight of 61.78 kg, body mass index (BMI) of 22.39, were enrolled. Five participants had a history of hypertension, four had a history of diabetes, and three had a history of hypertension and diabetes. According to regulations, medication was not discontinued during the study process. Moreover, no participant took antibiotics during the subject, nor had developed infection recently, and no other probiotics and yogurts were taken. None of these volunteers was vegetarian (Table 1).

**Table 1:** Baseline patient demographics and characteristics.

**Variable**	**C Group (*N* = 16)**	**P Group (*N* = 16)**	***P*-value**
Percentage of total enrollment, No. (%)	16 (50.00)	16 (50.00)	/
Male: female, *n*:*n* (%:%)	14:2 (87.50:12.50)	15:1 (93.75:6.25)	0.56
Age, year	53.50 (46.69–60.31)	48.19 (41.88–54.50)	0.23
Height, m	1.65 (1.62–1.68)	1.67 (1.64–1.71)	0.21
Weight, kg	61.50 (56.57–66.43)	62.06 (57.37–66.75)	0.86
Body mass index (BMI), kg/m^2^	22.72 (20.99–24.45)	22.06 (21.01–23.12)	0.50
**Past medical history**			
Hypertension, %	3 (18.75)	2 (12.50)	0.64
Diabetes, %	2 (12.50)	2 (12.50)	0.15
Gastrointestinal diseases history, %	3 (18.75)	2 (12.50)	0.64
Smoking history, %	2 (12.50)	3 (18.75)	0.64
Drinking history, %	3 (18.75)	2 (12.50)	1.00
Gastrointestinal reaction before bowel preparation, %	1 (6.25)	1 (18.20)	0.56
Gastrointestinal reaction after preparation, %	7 (43.75)	3 (18.75)	0.14

*C group, volunteers after treatment with placebo; P group, volunteers after treatment with probiotics*.

### Trial Protocol

The 32 volunteers were divided into two groups: placebo group (C group, *n* = 16) and probiotic group (P group, *n* = 16). Probiotic (*Bifidobacterium* Tetragenous viable Bacteria Tablets (SiLianKang), Hangzhou Grand Biologic Pharmaceutical Inc., Hangzhou, China. SFDA approval number: S20060010, containing >0.5 × 106 CFU/table *Bifidobacterium* infantis, >0.5 × 106 CFU/table *Lactobacillus acidophilus*, >0.5 × 106 CFU/table *Enterococcus faecalis*, and >0.5 × 105 CFU/table *Bacillus cereus*). Participants were suggested to eat porridge, noodles and other low-fiber diets the day before bowel preparation, and ate normally diets after colonoscopy. Antibiotics were forbidden during treatment process, as well as drinking and acrimony. All of them started taking placebo or probiotic preparations after colonoscopy for up to 5–7 days (three tablets and three times a day).

Participants took 2 L polyethylene glycol (PEG, SFDA approval number: H20020031, containing package A: 0.74 g potassium chloride and 1.68 g sodium bicarbonate, package B: 1.46 g sodium chloride and 5.68 g sodium sulfate, package C: 60 g polyethylene glycol 4,000) 4–5 h before colonoscopy and PEG should be completely taken within 1 h. After intravenous anesthesia with 1 ml lidocaine hydrochloride injection (SFDA approval number: H37021309), 1 ml nalbuphine hydrochloride injection (SFDA approval number: H20130127) and 20 ml propofol emulsion injection (SFDA approval number: H20051843), participants underwent colonoscopy. If participants were intolerant during colonoscopy, anesthetics were added as appropriate. Feces in 3 time pints (3 days before, the same day as the bowel preparation just before the colonoscopy, and 7 days after the process) were collected. The collected samples were stored in 50% glycerol (Cat#56-81-5; Sengon Biotech, China) and immediately stored at −80°C for further use.

### Total Bacterial Genomic DNA Extraction and High-Throughput Sequencing

A total of 96 fecal samples were collected, and the method of bead blasting combined with genomic DNA kit (Tiangen Biotech Co., Ltd., Beijing, China) was used to extract fecal microbial DNA (12). The concentration and purity of purified DNAs were determined via a spectrophotometer at 230 nm (A 230) and 260 nm (A 260) (NanoDrop; Thermo Fisher Scientific, Inc., Waltham, MA, USA). The V4 region of 16S rDNA gene in each sample was amplified with 515F/806R primer(515F, 5′-GCACCTAAYTGGGYDTAAAGNG-3′; 806R, 5′- TACNVGGGTATCTAATCC-3′), and PCR products were sequenced on IlluminaHiSeq 2000 platform (GenBank accession number PRJNA597277) (13).

### Data Analysis

To analyze the high-throughput sequencing data, Cutadapt (version 1.9.1, http://cutadapt.readthedocs.io/en/stable/), UCHIME algorithm http://www.drive5.com/usearch/manual/uchime_algo.html, UPARSE software package (version 7.0.100), QIIME software (version 1.9.1), QIIME software package (version 1.8.0) and SIMCA-P software (version 11.5; Umetrics; Sartorius Stedim Biotech, Malmö, Sweden) were used to determine the α diversity (within samples, indexes of observed OTUs, Chao1, Shannon, Simpson, ACE, and goods coverage) and β diversity (among samples, PCA, PCoA and NMDS) (14, 15).

Data are presented as means ± standard deviation (SD). Statistical analyses were performed using Prism software (version 7.0; GraphPad Software, San Diego, CA, USA) and SPSS 17.0 software (SPSS Inc., Chicago, IL, USA). Statistical significance was determined using one-way analysis of variance (ANOVA) followed by Tukey's multiple comparison test and F-tests. Error probabilities of *P* < 0.05 were considered statistically significant.

## Results

### Patients Baseline Characteristics

Between December 2018 and November 2019, 32 volunteers were enrolled into the placebo group (C group, 16 volunteers) and the probiotics group (P group, 16 volunteers), and their sex, age, BMI, baseline characteristics, past medical history and gastrointestinal reaction before and after bowel preparation were summarized in Table 1. There was no significant difference between C group and P group.

### Effect of Bowel Preparation on Intestinal Microbes

To explore whether bowel preparation can effect intestinal microorganisms, the V4 hypervariable region of bacteria was amplified using the 16S rDNA amplicon sequencing method from feces of 16 volunteers before (CB group), during (CM group) and after bowel preparation (CA group).

**Figure 1:**
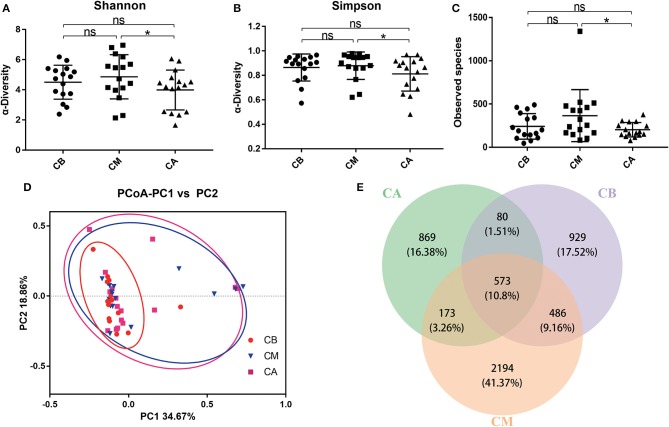
Effect of bowel preparation on intestinal microbiota. **(A)**, Shannon index; **(B)**, Simpson index; **(C)**, Observed species; **(D)**, PCoA of β diversity index; **(E)**, scalar-Venn representation. CB, control group 3 days before bowel preparation (*n* = 16); CM, control group during bowel preparation (*n* = 16); CA, control group 5–7 days after bowel preparation (*n* = 16). Data are presented as means ± SD. ns, *P* > 0.05; ^*^P < 0.05.

In Figures 1A–C, the Shannon index, Simpson index and Observed species indicated that the occurrence of bowel preparation slightly affected the α-diversity of the intestinal microbial community between the CB and CM groups, CB and CA groups, while significantly affected the microbial diversity between CM and CA groups (*P* < 0.05). And the principal coordinates analysis (PCoA) exhibited that the microbial diversity in CM group and CA group were different compared with that in CB group (Figure 1D). Additionally, the Venn index (Figure 1E) results indicated that there were 2,068, 3,426, and 1,695 OTUs in the CB, CM and CA groups, and their percentage of common OTUs were 27.71% (573/2,068), 16.73% (573/3,426) and 33.81% (573/1,695), respectively.

**Figure 2:**
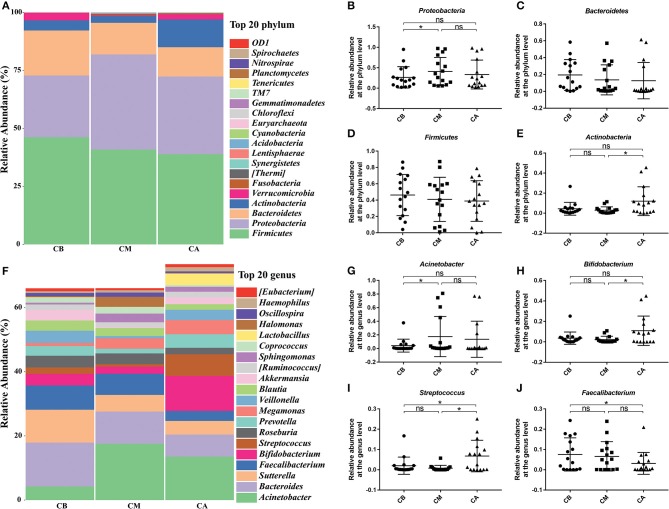
Effect of bowel preparation on microbial composition at phylum and genus levels. **(A)**, the relative abundance of intestinal microbiota at the phylum level; **(B)**, *Proteobacteria*; **(C)**, *Bacteroidetes*; **(D)**, *Firmicutes*; **(E)**, *Actinobacteria*. **(F)**, the relative abundance of intestinal microbiota at the genus level; **(G)**, *Acinetobacter*; **(H)**, *Bifidobacterium*; **(I)**, *Streptococcus*; **(J)**, *Faecalibacterium*. CB, control group 3 days before bowel preparation (*n* = 16); CM, control group during bowel preparation (*n* = 16); CA, control group 5–7 days after bowel preparation (*n* = 16). Data are presented as means ± SD. ns, *P* > 0.05; ^*^P < 0.05.

Furthermore, we further analyzed the dominant bacteria at the phylum level (Figures 2A–E), and found that *Proteobacteria, Bacteroidetes, Firmicutes*, and *Actinobacteria* were the predominant phyla in these 3 groups. The results revealed that bowel preparation increased the relative abundance of *Proteobacteria* (0.266 vs. 0.410) while decreased the relative abundance of *Actinobacteria* (0.044 vs. 0.029), and had slightly effect on the relative abundance of *Firmicutes* (0.462 vs. 0.408) and *Bacteroidetes* (0.194 vs. 0.136) compared with the CB group. Seven days after bowel preparation, the relative abundance of *Proteobacteria* decreased from 0.410 to 0.335. Strangely, the relative abundance of *Actinobacteria* increased from 0.029 to 0.119, while the relative abundance of *Firmicutes* (0.409 vs. 0.389) and *Bacteroidetes* (0.136 vs. 0.126) still showed a decreasing trend compared with the CM group. At the genus level (Figures 2F–J), during the bowel preparation, it was observed that the relative abundance of *Bacteroides* and *Acinetobacter* were dominant bacteria, the relative abundance of *Acinetobacter* (0.042 vs. 0.176) was significantly increased, and the relative abundance of *Streptococcus* (0.020 vs. 0.008), *Bifidobacterium* (0.036 vs. 0.022) and *Faecalibacterium* (0.075 vs. 0.065) was slightly altered compared with the CB group. Seven days after bowel preparation, however, there was a significant increase of the percentage of *Streptococcus* (0.007 vs. 0.068) and *Bifidobacterium* (0.022 vs. 0.109), and a decrease of the relative abundance of *Faecalibacterium* (0.065 vs. 0.031) and *Acinetobacter* (0.176 vs. 0.136) compared with the CM group.

### Effect of Probiotic Preparation on Intestinal Microbial Balance

To evaluate the effects of probiotics on intestinal microbiota of volunteers receiving bowel preparation, feces were collected before (PB group), during (PM group) and after bowel preparation (PA group) for 7 days (have taken probiotic preparation for 5–7 days).

**Figure 3:**
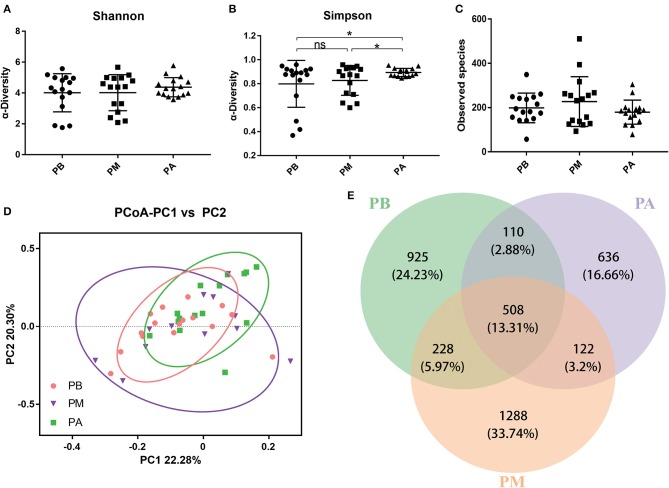
Effect of probiotics on intestinal microbiota. **(A)**, Shannon index; **(B)**, Simpson index; **(C)**, Observed species; **(D)**, PCoA of β diversity index; **(E)**, scalar-Venn representation. PB, probiotic group 3 days before bowel preparation (*n* = 16); PM, control group during bowel preparation (*n* = 16); PA, probiotic group 5–7 days after bowel preparation (Supplement the *Bifidobacterium* Tetragenous viable Bacteria Tablets after colonoscopy for up to 5–7 days, three tablets and three times a day) (*n* = 16). Data are presented as means ± SD. ns, *P* > 0.05; ^*^P < 0.05.

We observed that the bowel preparation had markedly affected the α-diversity on the Shannon index (Figure 3A) and Simpson index (Figure 3B, *P* < 0.05) of the microbial community between the PB and PM groups. Interestingly, the Observed species received an obvious increase after bowel preparation but an obvious reduction 7 days after the treatment (Figure 3C). Moreover, PCoA results indicated that taking probiotics greatly restored the disturbed microbiota to normal level in PM group and PA group (Figure 3D), and the common OTUs occupied 28.68% (508/1,771), 23.67% (508/2,146) and 36.92% (508/1,376) of the total OTUs in PB, PM and PA groups, respectively.

**Figure 4:**
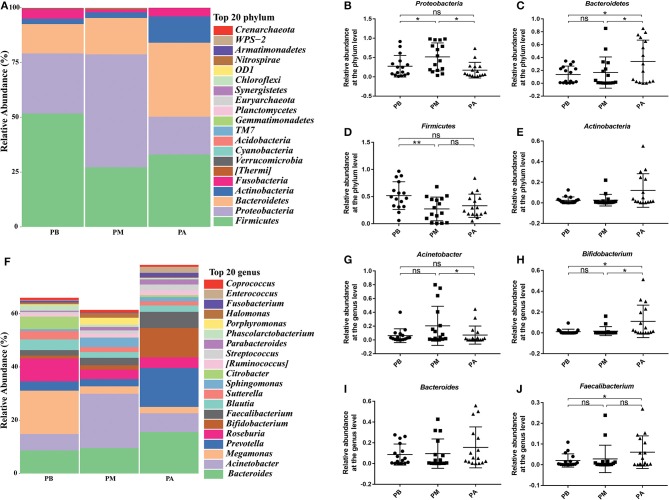
Effect of probiotics on microbial composition at phylum and genus levels. **(A)**, the relative abundance of intestinal microbiota at the phylum level; **(B)**, *Proteobacteria*; **(C)**, *Bacteroidetes*; **(D)**, *Firmicutes*; **(E)**, *Actinobacteria*. **(F)**, the relative abundance of intestinal microbiota at the genus level; **(G)**, *Acinetobacter*; **(H)**, *Bifidobacterium*; **(I)**, *Bacteroides*; **(J)**, *Faecalibacterium*. PB, probiotic group 3 days before bowel preparation (*n* = 16); PM, control group during bowel preparation (*n* = 16); PA, probiotic group 5–7 days after bowel preparation (Supplement the *Bifidobacterium* Tetragenous viable Bacteria Tablets after colonoscopy for up to 5–7 days, three tablets and three times a day) (*n* = 16). Data are presented as means ± SD. ns, *P* > 0.05; ^*^P < 0.05; ^**^P < 0.01.

Then, we further evaluated the effects of probiotic intervention on microbial composition, and found supplementation of probiotics significantly reduced *Proteobacteria* (0.515 vs. 0.173) and sharply increased the relative abundance of *Bacteroides* (0.166 vs. 0.338) in PM group compared with PA group at phylum level (*P* < 0.05). At the genus level, the supplemented probiotics had obviously reduced the percentage of *Acinetobacter* (0.204 vs. 0.071) in PM group compared with PA group, and significantly enhanced the percentage of *Bifidobacterium* (0.017 vs. 0.110), *Bacteroides* (0.095 vs. 0.155) and *Faecalibacterium* (0.028 vs. 0.060) in PM group compared with PA group (Figures 4F–J).

### The Microbial Changes Between Groups CA Group and PA Group

**Figure 5:**
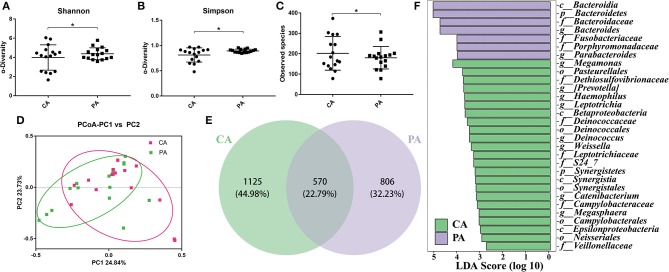
The microbial changes between groups CA and PA. **(A)**, Shannon index; **(B)**, Simpson index; **(C)**, Observed species; **(D)**, PCoA of β diversity index; **(E)**, scalar-Venn representation; **(F)**, Lefse index. CA, control group 5–7 days after bowel preparation (n=16); PA, probiotic group 5–7 days after bowel preparation (Supplement the *Bifidobacterium* Tetragenous viable Bacteria Tablets after colonoscopy for up to 5–7 days, three tablets and three times a day) (*n* = 16). Data are presented as means ± SD. ^*^P < 0.05.

To better understand the effect of probiotics on bowel preparation, we compared the microbial diversity between volunteers in groups CA and PA. As shown in Figures 5A–C, supplementation of probiotics had markedly enhanced the Shannon index and Simpson index (*P* < 0.05), while decreased the observed species. The PCoA results indicated that samples in CA group and PA group scattered far away from each other (Figure 5D). There were 1,695 and 1,376 OTUs in the CA group and PA group, and the common OTU number was 570 (Figure 5E). In addition, the Lefse analysis demonstrated that *Bacteroidia* (at class), *Bacteroidetes* (at phylum), *Bacteroidaceae* (at family), *Bacteroides* (at genus), *Fusobacteriaceae* (at family), *Porphyromonadaceae* (at family), and *Parabacteroides* (at genus) were significantly higher in the PA group than in the CA group (Figure 5F).

**Figure 6:**
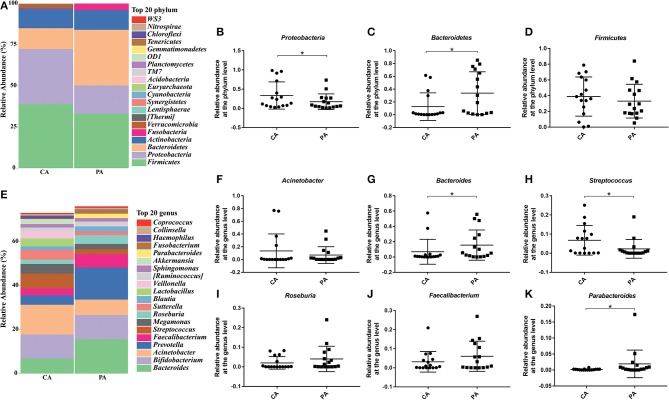
Effect of probiotics on microbial composition between groups CA and PA at phylum and genus levels. **(A)**, the relative abundance of intestinal microbiota at the phylum level; **(B)**, *Proteobacteria*; **(C)**, *Bacteroidetes*; **(D)**, *Firmicutes*. **(E)**, the relative abundance of intestinal microbiota at the genus level; **(F)**, *Acinetobacter*; **(G)**, *Bacteroides*; **(H)**, *Streptococcus*; **(I)**, *Roseburia*; **(J)**, *Faecalibacterium*; **(K)**, *Parabacteroides*. CA, control group 5–7 days after bowel preparation (*n* = 16); PA, probiotic group 5–7 days after bowel preparation (Supplement the *Bifidobacterium* Tetragenous viable Bacteria Tablets after colonoscopy for up to 5–7 days, three tablets and three times a day) (*n* = 16). Data are presented as means ± SD. ^*^P < 0.05.

Then, specific bacteria in CA group and PA group were compared. Supplementation of probiotics markedly enriched the percentage of *Bacteroidetes* (0.126 vs. 0.338), while reduced the percentage of *Proteobacteria* (0.335 vs. 0.173) and *Firmicutes* (0.389 vs. 0.330) compared with the PA group at the phylum level (*P* < 0.05). At genus level (Figures 6E–K), the supplementation of probiotics decreased the relative abundance of *Acinetobacter* (0.136 vs. 0.071) and *Streptococcus* (0.068 vs. 0.023), while increased the relative abundance of *Bacteroides* (0.068 vs. 0.155), *Roseburia* (0.02 vs. 0.04), *Faecalibacterium* (0.031 vs. 0.060) and *Parabacteroides* (0.16 vs. 1.92%).

## Discussion

Bowel cleaning is necessary during colonoscopy, and its long-time use of safety makes people ignore its negative impact on intestinal microorganisms (16). As we know, colonic microorganisms are the basis for promoting normal mammalian physiological functions, including angiogenesis, metabolism, digestion and immune system development (17). What's more, various diseases, including obesity, type 2 diabetes, colorectal cancer and inflammatory bowel disease will occur when intestinal microbiota is out of balance (8, 9, 18).

The main components of normal intestinal microecosystem are obligate anaerobes (*Bacteroidetes* and *Firmicutes*), and facultative anaerobes (such as *Proteobacteria*) usually only account for a small proportion, and the imbalance of intestinal microbiota is often caused by increasing number of facultative anaerobes (19). The bowel preparation can bring a large amount of oxygen into the intestinal cavity, damage the anaerobic environment of the intestinal cavity and provide a good growth environment for facultative anaerobic or aerobic bacteria. Previous studies and the present work, likewise, demonstrated that bowel preparation had greatly increased the abundance of *Proteobacteria* [Figures 2, 3; (19)].

In this study, we found that the bowel preparation significantly decreased phyla *Bacteroidetes* and *Firmicutes* (Figure 2), and taking probiotics had little effect on the phylum *Firmicutes*, while greatly increased the abundance of *Bacteroidetes* (Figure 4). Previous studies had shown that the increase of the abundance of *Firmicutes* and the decrease of the abundance of *Bacteroidetes* were closely related to unhealthy conditions. Therefore, the increase of *Firmicutes*/*Bacteroidetes* may pose a potential risk to patient's health (20–22). In the present study, we found that the ratio of *Firmicutes*/ *Bacteroidetes* in CA group (3.08) was higher than that in PA group (0.98), suggesting that taking probiotics could reduce the potential disease risks by bowel preparation. In addition, human body usually does not have the ability to degrade most of complex polysaccharides (the main component and main nutrition source of our daily diet) until reaching the colon, and *Bacteroidetes* play a vital role in degrading complex polysaccharides of cellulose, pectin and xylan, which can help people absorb more energy from the diet (23). Moreover, butyrate produced by *Bacteroidetes* plays an important role in maintaining the intestinal health of the host, exerting immunity and anti-tumor effect (24).

At genus level, supplementation of probiotics significantly reduced the abundance of *Acinetobacter* which is a common aerobic and gram-negative bacterium in nature, belonging to a vital pathogen causing hospital infection especially in patients with low immune function (*Acinetobacter baumannii*) (25). *Acinetobacter* had been listed as the third commonest human pathogen in the intensive care unit of South Korean hospitals, and its inherent drug resistance to a variety of antibiotics made it obtain determinants of drug resistance to various antibacterial drugs (26). Additionally, bowel preparation significantly reduced the level of *Bacteroides*, and probiotics obviously recovered its abundance. Studies had revealed that *Bacteroides* can reduce the intestinal oxygen level to promote the growth of strict anaerobes (23) and some strains of *Bifidobacterium* have been put into use as probiotics in food and medicine (10, 27). *Bacteroides* and *Bifidobacterium* can establish stable and long-term contact with host and benefit health of human body, can degrade dietary fiber into short-chain fatty acids (SCFAs), which provides energy source for cells, promotes barrier function and reduces the occurrence of inflammatory reactions (28, 29).

In the end, we compared the microbial diversity of CA and PA, and found that taking probiotics predominantly enhanced the abundance of beneficial bacteria such as *Roseburia* (mainly or only produces butyrate, which can reduce the level of inflammation in the whole, especially in the blood, further reduce the degree of atherosclerosis. It remains lower levels in people with cardiovascular diseases) (30, 31), *Faecalibacterium* (a symbiotic bacterium that widely exists in the gastrointestinal tract of animals and humans. It was significantly reduced in Crohn's patients, and might be used as a probiotic to treat Crohn's disease) (32–34) and *Parabacteroides* (can resist intestinal inflammation, the main metabolic end products are beneficial acetic acid and succinic acid, which are lower than the normal range in intestinal tract of patients with colitis) (35–37) at the genus level. Nevertheless, the abundance of harmful bacteria such as *Streptococcus* was strikingly reduced (Figures 5, 6). *Streptococcus* is a common opportunistic pathogen, including *Streptococcus pyogenes, Streptococcus viridans* and *Streptococcus pneumoniae*, which can cause purulent inflammation, endocarditis and septicaemia, further threaten human health and life (38, 39). Haenni et al. indicated that due to the widespread use of Tetracycline, Macrolide, and Lincosamide antibiotics in the global animal sector, the antibiotic resistance of *Streptococcus* zooepidermidis has emerged, leading to treatment failure (40, 41).

In the present study, we found that oral probiotics did alleviate the intestinal microbial disturbance caused by bowel preparation, greatly reduced the pathogens of *Proteobacteria* (at the phylum level), *Acinetobacter* (at the genus level), *Streptococcus* (at the genus level), and enhanced the probiotics of *Bacteroidetes* (at the phylum level), *Bacteroides* (at the genus level), *Roseburia* (at the genus level), *Faecalibacterium* (at the genus level) and *Parabacteroides* (at the genus level). Therefore, we have reasons to believe that supplement of probiotic preparations will accelerate the establishment of intestinal microbial balance after intestinal cleaning, suppress the growth of harmful bacteria and benefit the maintenance of intestinal health.

## Data Availability Statement

The datasets generated for this study can be found in the NCBI: GenBank accession number PRJNA597277.

## Ethics Statement

The studies involving human participants were reviewed and approved by Institutional Review Boards of the Second Affiliated Hospital of Nanchang University (Nanchang, China). The project has also been registered and approved by the China Clinical Trial Registration Center (ChiCTR1900022539). The patients/participants provided their written informed consent to participate in this study.

## Author Contributions

TC and XD designed the experiments, analyzed the data, and wrote the manuscript. CZ, HT, RY, ZL, YH, and KW performed the experiments. All authors discussed the results and commented on the final manuscript.

### Conflict of Interest

The authors declare that the research was conducted in the absence of any commercial or financial relationships that could be construed as a potential conflict of interest.

## References

[1] Kim MS Park J Park JH Kim HJ Jang HJ Joo HR. Does polyethylene glycol. (PEG) plus ascorbic acid induce more mucosal injuries than split-dose 4-L PEG during bowel preparation? Gut Liver. (2016) 10:237–43. 10.5009/gnl14439 26260754 PMC4780453

[2] Schoenfeld P Dominitz JA. No polyp left behind:defining bowel preparation adequacy to avoid missed polyps. Gastroenterology. (2016) 150:303–6. 10.1053/j.gastro.2015.12.024 26713765

[3] Parra-Blanco A Ruiz A Alvarez-Lobos M Amoros A Gana JC Ibanez P. Achieving the best bowel preparation for colonoscopy. World J Gastroentero. (2014) 20:17709–26. 10.3748/wjg.v20.i47.17709 25548470 PMC4273122

[4] Hofer U. Microbiome:bacterial imbalance in Crohn's disease. Nat Rev Microbiol. (2014) 12:312–3. 10.1038/nrmicro3255 24638106

[5] Rea K O'Mahony SM Dinan TG Cryan JF. The role of the gastrointestinal microbiota in visceral pain. Handb Exp Pharmacol. (2017) 239:269–87. 10.1007/164_2016_115 28035535

[6] Jalanka J Salonen A Salojarvi J Ritari J Immonen O Marciani L. Effects of bowel cleansing on the intestinal microbiota. Gut. (2015) 64:1562–8. 10.1136/gutjnl-2014-307240 25527456

[7] Bron PA Kleerebezem M Brummer RJ Cani PD Mercenier A MacDonald TT. Can probiotics modulate human disease by impacting intestinal barrier function? Brit J Nutr. (2017) 117:93–107. 10.1017/S0007114516004037 28102115 PMC5297585

[8] Tojo R Suarez A Clemente MG de los Reyes-Gavilan CG Margolles A Gueimonde M. Intestinal microbiota in health and disease:role of bifidobacteria in gut homeostasis. World J Gastroentero. (2014) 20:15163–76. 10.3748/wjg.v20.i41.15163 25386066 PMC4223251

[9] Nie P Li Z Wang Y Zhang Y Zhao M Luo J. Gut microbiome interventions in human health and diseases. Med Res Rev. (2019) 39:2286–313. 10.1002/med.21584 30994937

[10] Zheng C Chen T Wang Y Gao Y Kong Y Liu Z. A randomised trial of probiotics to reduce severity of physiological and microbial disorders induced by partial gastrectomy for patients with gastric cancer. J Cancer. (2019) 10:568–76. 10.7150/jca.29072 30719153 PMC6360416

[11] Jiang C Wang H Xia C Dong Q Chen E Qiu Y. A randomized, double-blind, placebo-controlled trial of probiotics to reduce the severity of oral mucositis induced by chemoradiotherapy for patients with nasopharyngeal carcinoma. Cancer. (2019) 125:1081–90. 10.1002/cncr.31907 30521105

[12] Meng F Chen T Wang X Wang X Wei H Tian P. Evaluation of the accuracy and sensitivity of highthroughput sequencing technology using known microbiota. Mol Med Rep. (2018) 17:408–13. 10.3892/mmr.2017.7849 29115413

[13] Liu Z Kong Y Gao Y Ren Y Zheng C Deng X. Revealing the interaction between intrauterine adhesion and vaginal microbiota using highthroughput sequencing. Mol Med Rep. (2019) 19:4167–74. 10.3892/mmr.2019.10092 30942434 PMC6472106

[14] Bolger AM Lohse M Usadel B. Trimmomatic:a flexible trimmer for illumina sequence data. Bioinformatics. (2014) 30:2114–20. 10.1093/bioinformatics/btu170 24695404 PMC4103590

[15] Edgar RC. UPARSE:highly accurate OTU sequences from microbial amplicon reads. Nat Methods. (2013) 10:996–8. 10.1038/nmeth.2604 23955772

[16] Moon W. Optimal and safe bowel preparation for colonoscopy. Clin Endosc. (2013) 46:219–23. 10.5946/ce.2013.46.3.219 23767029 PMC3678056

[17] Drago L Toscano M De Grandi R Casini V Pace F. Persisting changes of intestinal microbiota after bowel lavage and colonoscopy. Eur J Gastroenterol Hepatol. (2016) 28:532–7. 10.1097/MEG.0000000000000581 27015015

[18] Nelson MH Diven MA Huff LW Paulos CM. Harnessing the microbiome to enhance cancer immunotherapy. J Immunol Res. (2015) 2015:368736–48. 10.1155/2015/368736 26101781 PMC4458560

[19] Litvak Y Byndloss MX Tsolis RM Baumler AJ. Dysbiotic proteobacteria expansion:a microbial signature of epithelial dysfunction. Curr Opin Microbiol. (2017) 39:1–6. 10.1016/j.mib.2017.07.003 28783509

[20] Forbes JD Van Domselaar G Bernstein CN. The gut microbiota in immune-mediated inflammatory diseases. Front Microbiol. (2016) 7:1081. 10.3389/fmicb.2016.01081 27462309 PMC4939298

[21] Xue B Xie J Huang J Chen L Gao L Ou S. Plant polyphenols alter a pathway of energy metabolism by inhibiting fecal bacteroidetes and firmicutes *in vitro*. Food Funct. (2016) 7:1501–7. 10.1039/C5FO01438G 26882962

[22] Dwivedi M Ansarullah Radichev I Kemp EH. Alteration of immune-mechanisms by human microbiota and development and prevention of human diseases. J Immunol Res. (2017) 2017:6985256–8. 10.1155/2017/6985256 29445757 PMC5763106

[23] Wexler AG Goodman AL. An insider's perspective: Bacteroides as a window into the microbiome. Nat Microbiol. (2017) 2:17026–50. 10.1038/nmicrobiol.2017.26 28440278 PMC5679392

[24] Thomas F Hehemann JH Rebuffet E Czjzek M Michel G. Environmental and gut bacteroidetes:the food connection. Front Microbiol. (2011) 2:93. 10.3389/fmicb.2011.00093 21747801 PMC3129010

[25] Bergogne-Berezin E Towner KJ. *Acinetobacter* spp. as nosocomial pathogens:microbiological, clinical, and epidemiological features. Clin Microbiol Rev. (1996) 9:148–65. 10.1128/CMR.9.2.148-165.1996 8964033 PMC172888

[26] Gurung M Nam HM Tamang MD Chae MH Jang GC Jung SC. Prevalence and antimicrobial susceptibility of *Acinetobacter* from raw bulk tank milk in Korea. J Dairy Sci. (2013) 96:1997–2002. 10.3168/jds.2012-5965 23462164

[27] Allen AP Clarke G Cryan JF Quigley EMM Dinan TG. Bifidobacterium infantis 35624 and other probiotics in the management of irritable bowel syndrome. strain specificity, symptoms, and mechanisms. Curr Med Res Opin. (2017) 33:1349–51. 10.1080/03007995.2017.1322571 28436237

[28] Hamer HM Jonkers D Venema K Vanhoutvin S Troost FJ Brummer RJ. Review article:the role of butyrate on colonic function. Aliment Pharm Ther. (2008) 27:104–19. 10.1111/j.1365-2036.2007.03562.x 17973645

[29] Carlson JL Erickson JM Hess JM Gould TJ Slavin JL. Prebiotic dietary fiber and gut health:comparing the *in vitro* fermentations of beta-glucan, inulin and xylooligosaccharide. Nutrients. (2017) 9:1–17. 10.3390/nu9121361 29244718 PMC5748811

[30] Kasahara K Krautkramer KA Org E Romano KA Kerby RL Vivas EI. Interactions between *Roseburia intestinalis* and diet modulate atherogenesis in a murine model. Nat Microbiol. (2018) 3:1461–71. 10.1038/s41564-018-0272-x 30397344 PMC6280189

[31] Rohde KH Dyer DW. Mechanisms of iron acquisition by the human pathogens neisseria meningitidis and neisseria gonorrhoeae. Front Biosci Landmrk. (2003) 8:d1186–218. 10.2741/1133 12957813

[32] Benevides L Burman S Martin R Robert V Thomas M Miquel S. New insights into the diversity of the genus faecalibacterium. Front Microbiol. (2017) 8:1790. 10.3389/fmicb.2017.01790 28970823 PMC5609107

[33] Tap J Mondot S Levenez F Pelletier E Caron C Furet JP. Towards the human intestinal microbiota phylogenetic core. Environ Microbiol. (2009) 11:2574–84. 10.1111/j.1462-2920.2009.01982.x 19601958

[34] Sokol H Pigneur B Watterlot L Lakhdari O Bermudez-Humaran LG Gratadoux JJ. *Faecalibacterium* prausnitzii is an anti-inflammatory commensal bacterium identified by gut microbiota analysis of Crohn disease patients. Proc Natl Acad Sci USA. (2008) 105:16731–6. 10.1073/pnas.0804812105 18936492 PMC2575488

[35] Sinha R Ahn J Sampson JN Shi J Yu G Xiong X. Fecal microbiota, fecal metabolome, and colorectal cancer interrelations. PLoS ONE. (2016) 11:e0152126. 10.1371/journal.pone.0152126 27015276 PMC4807824

[36] Gao B Wang R Peng Y Li X. Effects of a homogeneous polysaccharide from Sijunzi decoction on human intestinal microbes and short chain fatty acids *in vitro*. J Ethnopharmacol. (2018) 224:465–73. 10.1016/j.jep.2018.06.006 29890316

[37] Gomez-Arango LF Barrett HL Wilkinson SA Callaway LK McIntyre HD Morrison M. Low dietary fiber intake increases collinsella abundance in the gut microbiota of overweight and obese pregnant women. Gut Microbes. (2018) 9:189–201. 10.1080/19490976.2017.1406584 29144833 PMC6219589

[38] Nguyen CT Park SS Rhee DK. Stress responses in *Streptococcus* species and their effects on the host. J Microbiol. (2015) 53:741–9. 10.1007/s12275-015-5432-6 26502957

[39] Turner AG Ong CY Walker MJ Djoko KY McEwan AG. Transition metal homeostasis in *Streptococcus* pyogenes and *Streptococcus pneumoniae*. Adv Microb Physiol. (2017) 70:123–91. 10.1016/bs.ampbs.2017.01.002 28528647

[40] Baracco GJ. Infections caused by group C and G *Streptococcus*. (*Streptococcus dysgalactiae* subsp. equisimilis and others):epidemiological and clinical aspects. Microbiol Spectr. (2019) 7:1–11. 10.1128/microbiolspec.GPP3-0016-2018 30977463 PMC11590429

[41] Haenni M Lupo A Madec JY. Antimicrobial resistance in *Streptococcus* spp. Microbiol Spectr. (2018) 6:1–25. 10.1128/microbiolspec.ARBA-0008-2017 29600772 PMC11633561

